# Microbicides for Topical HIV Immunoprophylaxis: Current Status and Future Prospects

**DOI:** 10.3390/ph17060668

**Published:** 2024-05-22

**Authors:** Yury V. Zhernov, Vladislava O. Petrova, Mark Y. Simanduyev, Denis V. Shcherbakov, Roman V. Polibin, Oleg V. Mitrokhin, Artem A. Basov, Nadezhda N. Zabroda, Sonya O. Vysochanskaya, Ezzulddin Al-khaleefa, Kamilla R. Pashayeva, Narmina Yu. Feyziyeva

**Affiliations:** 1 Department of General Hygiene, F. Erismann Institute of Public Health, I.M. Sechenov First Moscow State Medical University (Sechenov University), 119435 Moscow, Russia; 2 A.N. Sysin Research Institute of Human Ecology and Environmental Hygiene, Centre for Strategic Planning and Management of Biomedical Health Risks of the Federal Medical and Biological Agency, 119435 Moscow, Russia; 3 Fomin Clinic, 119192 Moscow, Russia; 4 The Baku Branch, I.M. Sechenov First Moscow State University (Sechenov University), Baku AZ1141, Azerbaijan; 5 Department of Public Health and Healthcare, Omsk State Medical University, 644099 Omsk, Russia; 6 Department of Epidemiology and Evidence-Based Medicine, F. Erismann Institute of Public Health, I.M. Sechenov First Moscow State Medical University (Sechenov University), 119435 Moscow, Russia; 7 Diphtheria and Pertussis Surveillance Laboratory, G.N. Gabrichevsky Research Institute for Epidemiology and Microbiology, 125212 Moscow, Russia

**Keywords:** HIV, AIDS, human immunodeficiency virus, acquired immunodeficiency syndrome, mucosal immunity, microbicides, pre-exposure prophylaxis

## Abstract

Microbicides, which are classified as topical antiseptic agents, are a revolutionary advancement in HIV prevention aimed to prevent the entry of infectious agents into the human body, thus stopping the sexual transmission of HIV and other sexually transmitted diseases. Microbicides represent the promise of a new age in preventive measures against one of the world’s most pressing health challenges. In addition to their direct antiviral effects during HIV transmission, microbicides also influence vaginal mucosal immunity. This article reviews microbicides by presenting different drug classifications and highlighting significant representatives from each group. It also explains their mechanisms of action and presents information about vaginal mucosal immune responses, emphasizing the critical role they play in responding to HIV during sexual transmission. The article discusses the following groups of microbicides: surfactants or membrane disruptors, vaginal milieu protectors, anionic polymers, dendrimers, carbohydrate-binding proteins, HIV replication inhibitors (reverse transcriptase inhibitors), and multi-purpose prevention technologies, which combine protection against HIV, other sexually transmitted diseases, and contraception. For each chemical compound, the article provides a brief overview of relevant preclinical and clinical research, emphasizing their potential as microbicides. The article offers insights into the multifaceted impact of microbicides, which signify a pivotal step forward in the pursuit of effective and accessible pre-exposure prophylaxis (PrEP).

## 1. Introduction

Based on the latest data form 2022, the number of people currently suffering from the human immunodeficiency virus (HIV) has reached 39 million worldwide. Despite the measures taken to prevent the spread of HIV, more than a million people are being infected annually. Ever since acquired immunodeficiency syndrome (AIDS) was first clinically observed, it has remained one of the main problems humanity has faced. In 2022, approximately 630,000 people died from AIDS-related illnesses. Moreover, mortality rates are remarkably higher for young adults in developing countries, reaching the highest numbers in sub-Saharan Africa [1,2].

Currently, 86% of all HIV infected people know their HIV status, and only 89% of them receive treatment. Considering the bleak statistics, slowing the spread of HIV and increasing the life expectancy should be the main intention. This, in turn, can be achieved through raising public awareness of HIV prevention methods, thus increasing the effectiveness and universal availability of both HIV diagnostics and active antiretroviral therapy [1,2,3,4,5].

Some studies indicate that the prophylaxis of HIV infection generates substantial economic benefits compared to those of therapy. Preventive measures can be implemented on different levels, including condom usage, voluntary medical male circumcision (VMMC), and pre-exposure prophylaxis (PrEP). Additionally, microbicides can be considered potential drugs used to combat the spread of HIV infection [6,7,8,9].

This review involves the description of some microbicides’ action mechanisms, which can help us understand their evidence base as we enter a new era of HIV prophylaxis.

## 2. Materials and Methods

**Figure 1:**
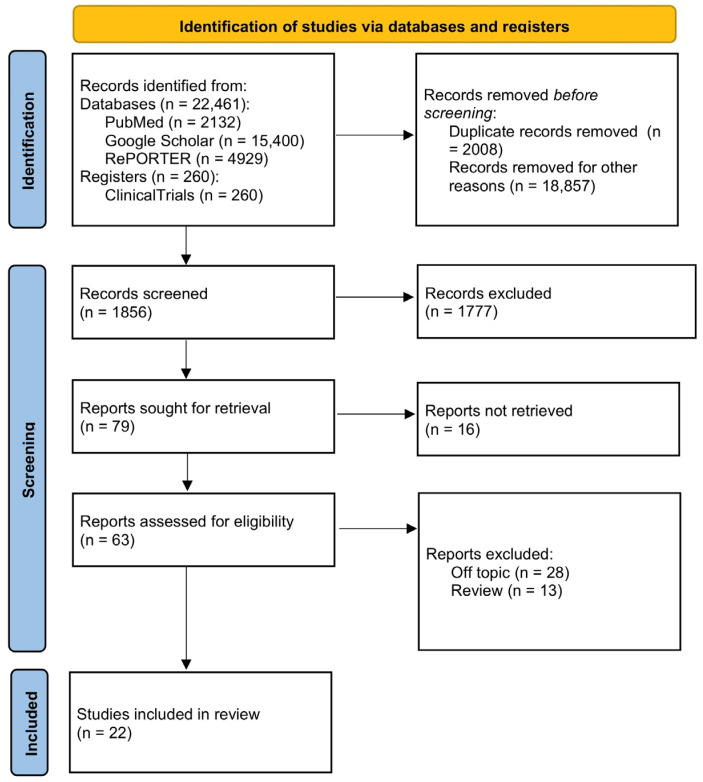
PRISMA flow diagram illustrating searches of databases and registers.

In this review, we collected information about mucosal immunity and microbicides from different groups. The aim was to describe the current state of microbicide research, highlight new drugs for PrEP (pre-exposure prophylaxis), and show where they are in clinical development and how effective their use is. We used a nonsystematic approach to search for information over a long period of time, starting with the earliest groups of microbicides and progressing to new prospective drugs that are undergoing preclinical and clinical trials. To accomplish this, we used databases such as PubMed, Google Scholar, RePORTER, and registers such as ClinicalTrials. We searched by keywords such as “HIV”, “microbicide”, and “prophylaxis”, and by product name. We selected randomized controlled trials and projects for this review using different combinations of these keywords. Inclusion criteria included up-to-date clinical and randomized controlled trials on different groups of microbicides used for topical immunoprophylaxis. We excluded review articles based on clinical trials, as well as those that contained off-topic data. All the aforementioned information is shown in Figure 1.

We outlined the main findings from the articles regarding the effectiveness of existing and newly developed microbicides for the most vulnerable populations. We categorized microbicides according to their mechanisms of action and the stage of development they currently exist at. The efficacy of each microbicide, as well as that of a placebo, was compared. Based on this information, we can identify the most promising areas for developing new microbicides and their potential uses.

## 3. Local Immune Response and Mucous Membranes

Most pathogens enter the body through mucous membranes, usually during ingestion, inhalation, or sexual contact, thus overcoming not only the physical barrier, but also the immune response organized by the mucosa-associated lymphoid tissue (MALT). In order to better understand the mechanisms of mucosal protection, let us take a closer look at the anatomy of the female reproductive tract (FRT). The lower FRT is covered by a thick, stratified squamous epithelium. In contrast, the upper FRT is lined with simple columnar epithelium. The transformation area between two tissues contains numerous CD4+ T cells and antigen-presenting cells [10,11]. Most of the ways in which HIV enters the lower FRT involve the breach of the mucosal surface during sexual activity, ulceration caused by other sexually transmitted infections, or the direct infection of CD4+ T cells or macrophages within the epithelium. It is noteworthy that studies suggest anal intercourse to be a more significant risk factor for HIV-1 acquisition than vaginal intercourse [12]. This is explained by the presence of CD4+ T-cells which are highly susceptible to HIV in the distal gastrointestinal tract and their rapid depletion after the infection, so it makes sense why diarrhea and wasting syndromes are some of the most common manifestations of the disease [13].

**Figure 2:**
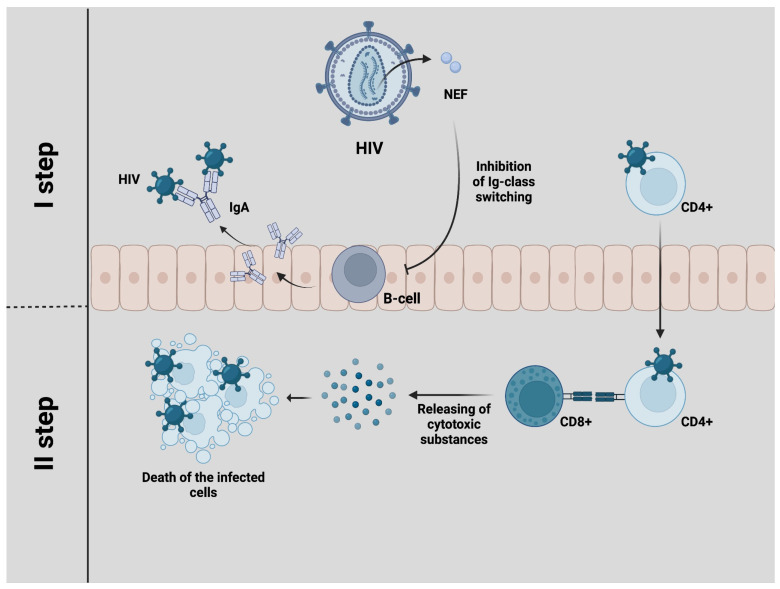
In the first step of selection, the virus genotype is chosen. B cells switch their class to IgA, capturing and neutralizing viral particles not linked to cells. On the other hand, HIV’s Nef protein impedes the immunoglobulin class-switching process, limiting the effectiveness of humoral immunity. The second step involves the selection of HIV particles associated with cells. CD8+ cells identify infected CD4+ cells and eliminate them by releasing cytotoxic substances.

The process of overcoming the mucosal barrier by HIV infection includes several steps. Firstly, the selection of the virus’s genotype occurs; therefore, not all virions will be able to penetrate through the mucous membranes. For instance, B cells, upon activation by mucosal dendritic cells, switch their class to IgA, which can capture and inactivate viral particles that are not associated with cells [14]. Unfortunately, HIV possesses various ways of suppressing the first line of mucosal defense. One hypothesis of an impaired humoral response relates to the impact of HIV-1 Nef on B-cell signaling and the inhibition of immunoglobulin class switching. This proposed mechanism involves the creation of actin-driven cellular conduits, which allows Nef to transit from infected macrophages and dendritic cells to uninfected germinal center B cells and accumulate there, which leads to diminished expression of activation-induced cytidine deaminase, an essential enzyme for class switching. This process is thought to interfere with the normal functioning of B cells and their ability to mount an effective immune response [15,16] (Figure 2).

After transmission of HIV to CD4+ cells, the next step of virus selection develops, in which the main role belongs to CD8+ cells, which must recognize infected cells and destroy them through the release of cytotoxic substances. However, upon close examination, it has been observed that gastrointestinal CD8+ T cells have notably lower levels of perforin and granzyme B compared to their counterparts in the blood [17]. Furthermore, two crucial transcription factors, whose expression is required for perforin expression and cytotoxicity, T-bet and Eomesodermin, are weakly expressed by rectal CD8+ T cells. Additionally, the HIV protein Nef limits the detection of infected cells by cytotoxic T lymphocytes through the selective downregulation of both HLA-A and HLA-B expression, reducing the presentation of viral epitopes on the cell surface [18]. Restriction of the cytotoxic response might prove beneficial to the host by limiting mucosal T-cell depletion and tissue damage. On the other hand, the inability to completely eliminate HIV-infected cells results in the failure of the second step of virus selection [19].

## 4. Microbicides

Microbicides represent a potential intervention strategy for preventing HIV transmission. When applied in the vagina or rectum, these medications work to inhibit the penetration of infectious agents affecting the components of mucosal immunity.

Almost three decades ago, the concept of a topical microbicide to protect against HIV was proposed. This idea was driven by the goal of providing women with protection that they can control, as the majority of prevention methods available to females are subject to the control of their male partners [20]. Along with effective vaccines, microbicides (topical preexposure prophylaxis (PrEP)) and oral PrEP have the potential to accomplish this goal. Both can also serve to protect both men and women from HIV transmission during unprotected anal intercourse [21].

Modern microbicides can be divided into fast-acting drugs, designed to be applied within 1 h before or after coitus, and drugs used to deliver the active component over a prolonged period of time. The first type includes various forms: gels, creams, ointments, lotions, tablets, aerosols, rinses, and application films. These types of microbicides offer the benefit of easy application, but their disadvantage is the brief duration of the active component’s effectiveness due to its rapid removal from the epithelial surface. Prolonged drugs include microbicides in forms of contraceptive sponges, intravaginal rings (IVR), uterine (cervical) caps, or enclosed in vaginal or rectal suppositories. They maintain the virucidal concentration of the active component over a greater period of time, but also require mechanical installation, for which gynecological intervention can be used [8,21].

The following groups of microbicides can be distinguished: surfactants or membrane disruptors; vaginal milieu protectors; anionic polymers; dendrimers; carbohydrate-binding proteins; and HIV replication inhibitors (reverse transcriptase inhibitors).

Multi-purpose prevention technologies (MPTs) deserve special attention—these are means that provide a combination of HIV prevention, prevention of a number of sexually transmitted diseases, and contraception.

For the most part, and this is evident from the research currently underway, these are advanced microbicides. Often, this is a hormonal drug that acts as a contraceptive and an antiretroviral drug. Among the MPTs being developed, it is worth noting the presence of an implant form, which is injected subcutaneously and gradually releases the active substance.

### 4.1. Surfactants/Membrane Disruptors

Surfactants were among the first compounds to undergo clinical evaluation as topical microbicides. Well-known representatives of the first generation include nonoxynol-9 (N-9), a mixture of cetyl betaine and myristamine oxide (1.0% C31G; SAVVY), and sodium lauryl sulfate (SLS). Their action mechanism is non-specific and involves the disruption of cellular and microbial membranes, which offers contraceptive activity against a wide range of pathogens. However, this could also be potentially toxic to host cells, leading to destruction of the epithelium, vaginal irritation, inflammation, tissue infiltration by host immune cells, and alterations in vaginal flora, consequently increasing the risk of HIV infection.

The first microbicide to be formally tested for its efficacy in preventing HIV transmission was N-9. A phase II efficacy trial with COL-1492, a N-9 vaginal gel in female sex workers revealed that applying it more than three times per working day doubled the risk of HIV infection compared to placebo users [22]. Another phase III clinical trial represented that the use of the N-9 vaginal film did not reduce the rate of new HIV infections [23]. Both of these results led to the conclusion that this can no longer be regarded as an effective method for preventing HIV.

C31G, showing broad anti-infective activity against many bacterial and viral STD pathogens, was tested in different safety trials and was advanced to a phase III clinical trial in Africa (Ghana and Nigeria) [24,25]. The Nigerian trial with SAVVY vaginal gel represented no significant differences among participants receiving SAVVY compared with those receiving a placebo [26].

Sodium lauryl sulfate (SLS) was represented as a potent inhibitor of the infectivity of several enveloped (HIV-1, Herpes simplex viruses, Semliki Forest virus) and nonenveloped (papillomaviruses, reovirus, rotavirus and poliovirus) viruses because of its protein-denaturing potency [27]. It was designed to function like an “invisible condom”, able to coat the vaginal wall in liquid form at room temperature and subsequently transition into a gel at body temperature. Although the results of a phase II study of 200 women in Cameroon have been pending, the interest in the development of surfactants, indicating a narrow margin between effectiveness and safety, has waned.

### 4.2. Vaginal Milieu Protectors

Vaginal milieu protectors, another broad group of microbicides, function to preserve the acidic environment within the vaginal canal at a pH level of 4, which has been shown to inactivate HIV [28]. The microbicides of this category either directly acidify or enhance the production of lactobacilli. They also serve as spermicides because the presence of semen neutralizes the baseline acidity of the vagina, thereby facilitating the penetration of the virus.

Carbopol 974P (BufferGel) is one of the modern representatives of microbicides and a polymer of acrylic acid that can significantly reduce the acidity of the vaginal environment. This, in turn, demonstrates virucidal activity against HIV, as confirmed through in vitro testing [29]. According to clinical studies conducted on female volunteers in India, Thailand, Malawi, and Zimbabwe, BufferGel has been proven to be safe and well tolerated by the cervicovaginal epithelium. Notably, there was a reported decrease in the prevalence of bacterial vaginosis after the first week of product use [30]. In 2009, the US National Institute of Allergy and Infectious Diseases (NIAID) discovered that repeated use of Buffer Gel led to the suppression of innate immunity. This finding prevented the product from entering the pharmaceutical market.

The Novel Intravaginal Ring, as a non-hormonal MPT from the Population Council, whose active ingredients are copper, zinc, and lactate, is also designed to optimize vaginal health and protect against STIs by increasing vaginal lactic acid concentrations. Copper IVR (Cu-IVR) from the University of California, which has a similar mechanism of action but does not offer protection against HIV, is also at an early preclinical stage. These drugs are also spermicides (they inhibit sperm motility).

Additionally, in the early 21st century, attempts were made by doctors to employ lactobacilli as microbicides for stabilizing vaginal pH—a remedy historically used for managing different dysbioses. However, due to the brief duration of therapeutic effects and the extended time required for lactobacilli colonies to flourish, research on microbicides based on non-genetically-modified *Lactobacillus* sp. was subsequently discontinued.

### 4.3. Entry Inhibitors

The group of microbicides based on anionic polymers and dendrimers includes substances such as carrageenans, naphthalene sulfonate polymers, cellulose sulfate, nanosized dendrimers, and humic acids. The action mechanism of this group involves the inhibition of viral fusion by binding to HIV surface proteins or to the receptors and co-receptors of target cells.

One of the first microbicides of this group was carrageenans, a family of linear sulfated polysaccharides derived from a seaweed extract. Carrageenans are similar in structure to heparan sulfate, which is used by many microorganisms as a biochemical receptor for initial attachment to the cell membrane. Thus, carrageenans act as decoy receptors for virus fusion [8]. However, a phase III study carried out on female volunteers in South Africa from 2004 to 2007 indicated that, while Carraguard gel was deemed safe over a 2-year period of use, the occurrence of new HIV infections was comparable in both the Carraguard and placebo groups. Thus, the study did not demonstrate a statistically significant impact of carrageenans on the sexual transmission of HIV infection [31].

Other polyanionic microbicides are naphthalene sulfonate polymers PIC 024-4 and PRO 2000. PRO 2000 competes with the V3 loop of gp120 HIV-1 envelope in binding with the target cell receptor CD4 [32]. Clinical trials of PRO 2000 vaginal gel conducted on female volunteers from South Africa, Tanzania, Uganda, and Zambia showed the safety of the drug; however, its effectiveness was not proven.

SPL7013 (or VivaGel^®^ or astodrimer sodium), belonging to macromolecules with a tree-like and regularly branching structure (dendrimers), is of great interest as it is the only medication that does not contain antiretroviral (ARV) agents, but still demonstrates excellent anti-HIV potential. This medication, which initially showed its effectiveness in treating bacterial vaginosis, has already completed phase 3 clinical trials (NCT01577537), and has been registered and approved for use in more than 55 countries. In addition, the company began producing lubricated condoms and nasal spray with the same active ingredient, which provides a moisturizing and protective barrier against respiratory viruses [33].

In the future, developments based on dendrimer-like substances, such as humic acids, which are found ubiquitously in nature, hold promise. These substances are hyperbranched polyelectrolytes, characterized by a network of polydisperse aromatic and aliphatic structures highly substituted by carboxyl and hydroxyl groups. Humic acids, known for their low toxicity and antioxidant activity, have synthetic analogs like HS-1500 (oxyhumate), which is obtained through hydroquinone polymerization. Research has shown their ability to inhibit HIV in MT-2 cells [34], with effective concentrations ranging from 50 to 300 ng/mL and a semi-toxic concentration of 600 µg/mL.

HS-1500’s proposed mechanism involves blocking the binding of the V3 loop of gp120 to the CD4 receptor on target cells. Studies by G.K. Joone et al. demonstrated that HS-1500 enhances the proliferative effect of phytohemagglutinin on lymphocytes from HIV-infected patients [35]. This stimulative effect was also observed when administering HS-1500 to HIV-positive patients at a dose of 4 g/day for 2 weeks. The observed proliferation stimulation correlated with increased production of interleukin (IL) 2 and expression of IL-2 receptors, along with a decrease in the amount of IL-10 under the influence of HS-1500.

Another promising group may be polysaccharide-binding substances, whose representatives belong to the group of natural peptides. The most prominent representatives are cyanovirin-N (CVN), a lectin compound derived from the cyanobacterium *Nostoc ellipsosporum*, and griffithsin (GRFT), derived from the red algae *Griffithsia*. Their mechanism of action involves the prevention of viral–host cell fusion by mannose residues on the surface of the virion. The effectiveness of these proteins against HIV infection when administered rectally and vaginally has been proven in preclinical studies [36]. The Population Council has begun to develop a vaginal insert based on this substance, and two studies are currently at the preclinical testing stage. For the purposes of this study, GRFT has been genetically modified to produce a more stable compound which is less prone to oxidation, called Q-GRFT. It will be safe and suitable for use by women of any age, as well as pregnant and lactating women [37].

Peptide microbicides are promising, as it is possible to integrate the gene encoding these peptides into any microorganism. At the U.S. National Institutes of Health (NIH), research is being conducted on the potential colonization of women’s vaginal microbiota with *Lactobacillus jensenii*, a bacteria containing an embedded gene producing the cyanovirin-N protein. Thus, this kind of microbicide will have multiple mechanisms of action: the ability to bind mannose residues on the virion surface and the ability to restore the normal vaginal flora.

### 4.4. HIV Replication Inhibitors

The mechanism of action of this group involves binding to the HIV-1 reverse Transcriptase (RT) enzyme, which is responsible for the conversion of viral RNA into proviral DNA, making viral replication impossible. One of the main distinguishing features of HIV replication inhibitors is that their active substances have already demonstrated efficacy in HIV therapeutics. There are nucleoside RT inhibitors (NRTIs), or nucleotide RT inhibitors, which mimic endogenous nucleotides and halt the elongation of the viral DNA chain within the target cell, and non-nucleoside RT inhibitors (NNRTIs), which in turn disrupt the binding of the substrate to the active site of the enzyme and have been demonstrated to bind irreversibly to RT [38]. The most famous representative of the NNRTI class is Dapivirine. Due to its irreversible binding and lipophilic properties, it may be active against both cell-free and cell-associated HIV. A vaginal gel based on dapivirine has demonstrated the ability to prevent HIV infection in humanized mice during preclinical trials [39]. The lead NRTI, in turn, is tenofovir, an adenosine monophosphate analogue. Tenofovir is converted into the active metabolite tenofovir diphosphate, which competes with deoxycytidine 5′-triphosphate for HIV-1 reverse transcriptase. It was formulated as a 1.0% vaginal gel with a microbicide composition.

Clinical trials conducted by the Centre for the AIDS Programme of Research in South Africa (CAPRISA) in July 2010 showed that tenofovir gel was 39% effective [40]. The results of third-phase (FACTS-001) clinical trials indicated that peri-coital tenofovir did not prevent HIV-1 acquisition among young women at risk for HIV infection in South Africa. Microbicides from this group play a crucial role in drugs designed for pre-exposure prophylaxis, which are currently undergoing both clinical and preclinical trials.

#### 4.4.1. Clinical Trials of Topical and Oral Pre-Exposure Prophylaxis (PrEP)

In introducing drugs designed for topical pre-exposure prophylaxis (PrEP) which are currently undergoing clinical trials, it is essential to highlight the diverse intravaginal rings (IVRS) as notable candidates in this emerging landscape. IVRS containing only tenofovir (TFV) or TFV plus levonorgestrel (LNG), which is a contraceptive hormone, have undergone several Phase I trials conducted by CONRAD in 2016 (NCT02235662) and 2019 (NCT03279120), respectively, and have successfully met all performance benchmarks. Currently, the NCT03255915 study of intravenous administration (TDF-FTC pod-IVR) is ongoing, but participants have not been recruited yet. A Phase-IIa trial conducted in Kenya (NCT03762382) showed that TFV/LNG and TFV-containing IVRS did not adversely affect the genital microbiota and are therefore safe to use [41].

Another type of IVRS includes those containing dapivirine, an NNRTI (non-nucleoside reverse transcriptase inhibitor), which are in their final stages of development. The completed Phase 3b trial by the Microbicide Trials Network (MTN 043) affirms the safety of monthly DPV (dapivirine)-containing IVRS, particularly when used by breastfeeding women. It is worth noting that DPV IVRs were approved for women by the South African Health Products Regulatory Agency (SAHPRA) for women in March 2022. This was immediately followed by an official WHO recommendation being issued. IVRs containing DPV/LNG, which are released over a period of 90 days, underwent Phase I trials, resulting in encouraging outcomes, supporting further development of the DPV/LNG IVR. Additionally, it is worth noting that fast-dissolving inserts are applicable for both vaginal and rectal use. They incorporate tenofovir alafenamide (TAF), a tenofovir prodrug, and elvitegravir (EVG), which is an HIV-1 integrase strand transfer inhibitor. The latter prevents the integration of HIV-1 DNA into the host DNA, thereby blocking the formation of the HIV-1 provirus [20,41,42]

These inserts have shown positive results in non-human primates, whether they are inserted vaginally hours before or after exposure to SHIV (simian human immunodeficiency virus). Additionally, in phase I trials (CONRAD 146 and MTN-039), the inserts were assessed for safety, pharmacokinetics, and pharmacodynamics. CONRAD 146 was a completed study that evaluated the TAF/EVG vaginal insert, while MTN-039 was a study focused on the rectal administration of the same, and its results are expected soon. Additionally, a placebo version of this insert was assessed in two global studies: the Quatro study comparing four vaginal forms in African women and the MTN-035 (DESIRE) study comparing three rectal forms. The next step involves bringing this drug to a comprehensive multi-dose safety and pharmacokinetic study [43,44].

It is necessary to mention the means being developed for HIV monoprophylaxis. For example, MK-8527, a new nucleoside reverse transcriptase translocation inhibitor, is being developed by Merck. This drug will be used as a once-weekly drug for treatment and as a monthly drug for PrEP. Merck had hoped that islatravir could be developed as a weekly or monthly antiretroviral, but at the higher doses needed for long-acting dosing, islatravir caused loss of lymphocytes. Currently, there are two trials of the drug underway. These are the MK-8527 Single-Dose Trial in HIV-1 Infected Participants (MK-8527-004) under registration number NCT05494736, taking place in Romania and South Africa, and the Safety and Pharmacokinetic Study of Oral MK-8527 QM in Participants at Low-Risk for HIV-1 Infection (MK-8527-007) under registration number NCT06045507, currently recruiting in Israel and the USA.

The first study evaluated the safety, tolerability, pharmacokinetics, and antiretroviral activity of MK-8527 in participants who had not previously received antiretroviral therapy with HIV-1 infection. The second study is a double-blind, placebo-controlled study designed to assess the safety, tolerability, and pharmacokinetics of oral MK-8527 taken once a month in participants at low risk of HIV-1 infection.

It is important to remember that PrEp drugs that have been approved by the Food and Drug Administration (FDA). There are two combined drugs from Gilead: Truvada and Descovy. Descovy contains two different medicines: emtricitabine and tenofovir alafenamide. Truvada contains two different active ingredients: emtricitabine and tenofovir disoproxil fumarate. Both are used for HIV PrEP to reduce the risk of HIV infection in adults and adolescents who weigh at least 77 lb (35 kg), are HIV-negative, and are at risk of contracting HIV. However, the Descovy drug for PrEP is not approved for use in females at risk of infection through vaginal sex, as this use has not been studied in clinical trials [45,46].

The company ViiV Healthcare is introducing Cabotegravir in two forms—tablets and long-acting injections. The Cabotegravir oral tablet (brand name: Vocabria) is used for the short-term treatment of HIV infection in adults and adolescents 12 years of age and older who weigh at least 77 lb (35 kg), as well as for short-term PrEP to reduce the risk of HIV infection in adults and adolescents who weigh at least 77 lb (35 kg), are HIV negative, and are at risk of contracting HIV. Long-acting injectable cabotegravir (brand name: Apretude) is used for HIV PrEP to reduce the risk of HIV infection in adults and adolescents who weigh at least 77 lb (35 kg), are HIV negative, and are at risk of contracting HIV [47].

Gilead Sciences presents Lenacapavir, approved by the FDA under the brand name Sunlenca for HIV treatment. Belonging to a group of capsid inhibitors, Lenacapavir can interfere with the HIV capsid, which protects the virus’s genetic material and replication enzymes. This interference leads to disruption at various stages of the viral life cycle, preventing HIV multiplication and reducing viral load. Lenacapavir is also under investigation as a preventive measure in ongoing studies (NCT04994509 in South Africa and Uganda, and NCT04925752 in the United States, Brazil, Puerto Rico, and South Africa), with Phase 3 results pending [48]. Ongoing clinical trials are shown in Table 1.

#### 4.4.2. Drugs in Preclinical and Early Clinical Phases

There are quite a few combined drugs undergoing preclinical and early-stage clinical development that hold potential for progressing into subsequent phases of clinical trials. We would have an easier time categorizing these drugs according to their hormonal classification. Drugs of the hormonal type typically consist of a hormonal contraceptive (LNG, EE, Etonogestrel or Norelgestromin) combined with various antiretroviral drugs such as DPV, TDF, TFV, or Cabotegravir and Islatravir. As mentioned earlier, these combinations serve to prevent ovulation and inhibit the replication of HIV.

Another example of potential hormonal drugs involves combining contraceptive hormones with the stable analog of non-anti-retroviral anti-HIV lectins, QGRFT, which binds to the HIV envelope glycoprotein and prevents entry into target cells. Non-hormonal microbicides composed of various components primarily aim to bind or inactivate sperm, thereby preventing not only pregnancy but also transmission of HIV. Some are based on combinations of monoclonal antibodies like mAb 2C7, which mediates the killing of *Neisseria gonorrhoeae* or agglutinating sperm antibodies with antiretroviral drugs, specifically TDF. Others are represented by IVR, containing a fully human antibody (HCA) that has the ability to agglutinate sperm, incorporated into a sustained release delivery system, which effectively prevents both pregnancy and the transmission of HIV, but monoclonal antibodies are not the only option for future PrEP. There are other combinations of drugs, like QGRFT with organic acids formulated into fast-dissolving inserts, that lower vaginal pH in the presence of semen, rendering it inactive. Other possible drugs for rendering semen infertile without harming other cells are combinations of polyphenylene and carboxymethylene (PPCM). PPCM causes premature acrosome loss in sperm and inhibits hyaluronidase, preventing sperm from fertilizing an ovum [52].

Except for new combinations with familiar delivery systems, certain products have been introduced using innovative ones. This involves incorporating TFV/EFV nanoparticles (NPs) into a polymer film base (TFV/EFV NPs in film), enabling higher concentrations of both drugs in the genitals compared to their delivery in a liquid carrier [53]. The combined microbicides discussed here, featuring groundbreaking innovations, have promising prospects. Notably, these formulations are at various stages in clinical trials, ranging from early to advanced phases, and are nearing commercial viability. A significant number of candidates are undergoing clinical investigation, highlighting the potential of microbicides for future preventive applications.

As these products progress through research phases, it becomes increasingly clear that microbicides have the potential to play a critical role in preventive healthcare strategies [20,54,55,56,57]. Summary chapters are available in Table 2 and Table 3. The chemical structures of the mentioned microbicides are presented in Table 4.

## 5. Discussion

Microbicides play a crucial role in the fight against HIV/AIDS by providing an additional prevention tool, particularly for populations facing barriers to other existing methods such as condoms or oral PrEP. The urgent need for microbicides targeting HIV, particularly those designed for vaginal application, stems from several critical factors that underline the ongoing global efforts to curb the spread of HIV/AIDS. Globally, women bear a disproportionate burden of HIV infection, especially in sub-Saharan Africa, where they account for a majority of new infections. Biological factors, such as the higher susceptibility of vaginal tissues to HIV transmission compared to penile tissues, contribute to this disparity. Vaginal microbicides offer a targeted approach to address this vulnerability by providing a protective barrier at the site of transmission, potentially reducing the risk of HIV acquisition among women. While significant progress has been made in HIV prevention, existing strategies such as condoms and oral PrEP have limitations that underscore the need for additional options. Condoms require partner cooperation and may not be consistently used, while oral PrEP may be inaccessible or unsuitable for some individuals. Vaginal microbicides offer a complementary approach that can be used discreetly and independently, filling critical gaps in the prevention toolkit and bolstering overall HIV prevention efforts.

## 6. Conclusions

In conclusion, the field of microbicides holds great promise for the ongoing fight against HIV infection. Recently discovered, these compounds offer a fertile ground for potential breakthroughs with numerous unexplored and unsynthesized forms. We have examined diverse classes of microbicides, dissecting their benefits and drawbacks. Our focus has narrowed down the most promising groups of preventive agents, exploring their evolution through different phases of clinical trials. Microbicides, with their multifaceted nature, have moved from the early research stage and are now on the verge of being available. Within clinical trials, the extensive interest in them has become apparent, revealing the nuanced promise that these agents hold for preventing HIV. Despite challenges such as optimizing efficacy and wide distribution, the documented progress indicates a hopeful trajectory. Ongoing exploration and synthesis of microbicides fuel optimism for innovative solutions in HIV prevention, making this field an exciting frontier for further research and development in the global battle against HIV/AIDS.

## Figures and Tables

**Table 1:** Clinical trials of pre-exposure prophylaxis currently underway.

Product Name	Delivery Method	Product Developer (Location)	Active Ingredients	Hormonal Type	Useful Links	Registration Number
Dapivirine + Levonorgestrel IVR	Intravaginal Ring (IVR)	International Partnership for Microbicides (Silver Spring, MD, USA)	Dapivirine Levonorgestrel	Hormonal	[49,50]	NCT05041699
MK-8527	Oral tablet	Merck (Darmstadt, Germany)	MK-8527 new nucleoside reverse transcriptase translocation inhibitor	Non-hormonal	[51]	NCT05494736 NCT06045507
Lenacapavir	Injectables (Subcutaneus)	Gilead (Foster City, CA, USA)	Lenacapavir in combination with other antiretrovirals	Non-hormonal	[48]	NCT04994509 NCT04925752

**Table 2:** Antiretroviral drugs at the preclinical and early clinical stages and their mechanisms of action.

Product Developer (Location)	Active Ingredients	Hormonal Type	Mechanism of Action (Meaning)
Population Council (New York City, NY, USA) Oak Crest Institute of Science (Monrovia, CA, USA)	Etonogestrel Ethinyl Estradiol QGRFT	Hormonal	Ovulation inhibition (pregnancy); binding to HIV envelope glycoproteins to prevent entry of HIV into target cells (QGRFT)
University of North Carolina (Chapel Hill, NC, USA)	Dapivirine Levonorgestrel Pritelivir	Hormonal	Antiretroviral drug + hormonal contraceptive
Auritec Pharmaceuticals (Pasadena, CA, USA)	Acyclovir Dolutegravir Etonogestrel Ethinyl Estradiol Rilpivirine	Hormonal	Antiretroviral drug + hormonal contraceptive
University of North Carolina (Chapel Hill, NC, USA)	Etonogestrel Ethinyl Estradiol Islatravir (EFdA)	Hormonal	Antiretroviral drug + hormonal contraceptive
MassBiologics (Boston, MA, USA) Oak Crest Institute of Science (Monrovia, CA, USA) Planet Biotechnology, Inc. (Hayward, CA, USA) University of Massachusetts (Amherst, MA, USA)	Monoclonal Antibodies Tenofovir Disoproxil Fumarate (TDF)	Non-Contraceptive	mAb 2C7 mediates complement-dependent killing of Neisseria gonorrhoeae. TDF is NRTI targeting HIV infection.
Oak Crest Institute of Science (Monrovia, CA, USA) University of North Carolina (Chapel Hill, NC, USA)	Monoclonal Antibodies Tenofovir Disoproxil Fumarate (TDF)	Non-Hormonal	Nucleoside analogue reverse transcriptase inhibitor (NRTI) and sperm-agglutinating antibody
MUCCOMMUNE LLC (Morrisville, NC, USA)	HCA Monoclonal Antibodies VRC01 + N6	Non-Hormonal	Viral neutralisation (sustained release of antibodies for non-hormonal contraception and HIV prevention)
Population Council (New York City, NY, USA)	QGRFT Organic acids	Non-Hormonal	The selected organic acids will lower vaginal pH (pH = 3.5–4.5) in the presence of semen. The low pH inactivates sperm and bacteria. When inserted into the vagina, the FDI dissolves into a viscous gel delivering the active agents throughout the vaginal lumen to prevent sperm penetration. QGRFT is a potent non-antiretroviral (ARV) inhibitor of HIV. QGRFT binds to HIV envelope glycoproteins and prevents the entry of HIV into target cells.
Magee-Women’s Research Inst. and Fndn., University of Pittsburgh (Pittsburgh, PA, USA)	4′-Ethynyl-2-fluoro-2′-deoxyadenosine (EFdA) 4′-Ethynyl-2-fluoro-2′-deoxyadenosine prodrug (EFdA-P) Progestin	Hormonal	Nucleoside reverse transcriptase translocation inhibitor and progestin hormonal contraceptive
Magee-Women’s Research Inst. and Fndn., University of Pittsburgh (Pittsburgh, PA, USA)	Dapivirine Levonorgestrel	Hormonal	Antiretroviral drug + hormonal contraceptive
Inst. For Research and Innovation in Health (i3S), University of Porto (Porto, Portugal)	Efavirenz (EFV) Tenofovir (TFV)	Non-Contraceptive	Short-term (on-demand) protective film that dissolves on contact with vaginal fluids to deliver a combination of antiviral compounds
Research Triangle International (RTI) (Research Triangle Park, NC, USA)	Unspecified	Hormonal	Nucleoside reverse transcriptase inhibitor; ovulation suppression
PATH (Seattle, WA, USA) Queen’s University Belfast (Belfast, Northern Ireland, UK)	Norelgestromin ARV candidates under review	Hormonal	ARV + Progestin
CONRAD (Norfolk, VA, USA)	Cabotegravir Levonorgestrel	Hormonal	Integrase strand-transfer inhibitor; progestin
University of North Carolina (Chapel Hill, NC, USA)	Hormonal Contraceptive Antiretroviral (ARV)	Hormonal	Antiretroviral drug + hormonal contraceptive
Yaso Therapeutics (Frisco, TX, USA)	Polyphenylene Carboxymethylene (PPCM)	Non-Hormonal	PPCM prevents viral binding and fusion to a host cell by attaching to and blocking key viral binding sites. It has been demonstrated that PPCM binds gB on HSV-2 and gp120 on HIV. PPCM has also shown promise against the Ebola virus in vitro, as well as gonorrhea in vitro and in mice. PPCM renders sperm infertile without damaging other cells.

**Table 3:** Antiretroviral drugs at the preclinical and early clinical stages.

Product Name	Indications	Clinical Phase	Product Developer (Location)	Active Ingredients	Hormonal Type	Useful Links
**Intravaginal ring (IVR)**						
90-day Pod-type Etonogestrel/Ethinyl Estradiol/QGriffithsin (EEQ)	HIV, Pregnancy	Preclinical—Early (Pre1)	Population Council (New York City, NY, USA) Oak Crest Institute of Science (Monrovia, CA, USA)	Etonogestrel Ethinyl Estradiol QGRFT	Hormonal	[58]
Dapivirine + Pritelivir + Levonorgestrel 3D Printed IVR	HIV, Pregnancy, HSV-2	Preclinical—Early (Pre1)	University of North Carolina (Chapel Hill, NC, USA)	Dapivirine Levonorgestrel Pritelivir	Hormonal	[59]
Novel Intravaginal Ring as a Non-Hormonal Contraceptive Multipurpose Prevention Technology (MPT)	Bacterial Vaginosis (BV) Chlamydia Gonorrhea HIV HSV-2 Pregnancy	Pre-formulation, pre-Phase 1, Non-clinical	Population Council (New York City, NY, USA) Queen’s University Belfast (Belfast, Northern Ireland, UK) Weill Cornell Medical College (New York City, NY, USA)	Copper Zinc Lactide	Non-Hormonal	[60]
Islatravir (EFdA) + Etonogestrel/Ethinyl Estradiol 3D Printed IVR	HIV Pregnancy	Preclinical—Early (Pre1)	University of North Carolina (Chapel Hill, NC, USA)	Etonogestrel Ethinyl Estradiol Islatravir (EFdA)	Hormonal	[61]
**IVR. mAb**						
mAb 2C7 + TDF IVR	HIV, Gonorrhea	Preclinical—Advanced (Pre2)	MassBiologics (Boston, MA, USA) Oak Crest Institute of Science (Monrovia, CA, USA) Planet Biotechnology, Inc. (Hayward, CA, USA) University of Massachusetts (Amherst, MA, USA)	Monoclonal Antibodies Tenofovir Disoproxil Fumarate (TDF)	Non-Contraceptive	[62]
Novel mAb contraceptive + Tenofovir Disoproxil Fumarate (TDF) IVR	HIV, Pregnancy	Preclinical—Early (Pre1)	Oak Crest Institute of Science (Monrovia, CA, USA) University of North Carolina (Chapel Hill, NC, USA)	Monoclonal Antibodies Tenofovir Disoproxil Fumarate (TDF)	Non-Hormonal	[63]
Human Contraceptive Antibody (HCA) + VRC01 + N6 IVR	HIV, Pregnancy	Preclinical—Advanced (Pre2)	MUCCOMMUNE LLC (Morrisville, NC, USA)	HCA Monoclonal Antibodies VRC01 + N6	Non-Hormonal	[64]
Non-ARV/Non-Hormonal Multi-Purpose Vaginal Ring	HIV HPV HSV-1 HSV-2 Pregnancy	Pre-formulation	Oak Crest Institute of Science (Monrovia, CA, USA)	Unspecified	Non-Hormonal	[65]
**Fast-dissolving inserts (FDIs) (Vaginal)**						
Non-hormonal Contraceptive Multipurpose Prevention Technology (MPT) Containing Q-Griffithsin (QGRFT)	Bacterial Vaginosis (BV) Chlamydia Gonorrhea HIV HSV-2 Pregnancy	Preclinical—Early (Pre1)	Population Council (New York City, NY, USA)	QGRFT Organic acids	Non-Hormonal	[66]
Griffithsin (GRFT) Fast-Dissolving Insert (FDI)	HIV HPV HSV-1 HSV-2	Pre-formulation	Population Council (New York City, NY, USA)	Unspecified	Non-Contraceptive	[67]
**Films (Vaginal)**						
EFdA-P + Progestin Intravaginal Film	HIV, Pregnancy	Early Preclinical	Magee-Women’s Research Inst. and Fndn., University of Pittsburgh (Pittsburgh, PA, USA)	4′-Ethynyl-2-fluoro-2′-deoxyadenosine (EFdA) 4′-Ethynyl-2-fluoro-2′-deoxyadenosine prodrug (EFdA-P) Progestin	Hormonal	[68]
Dapivirine/Levonorgestrel Extended-Release Monthly Film	HIV, Pregnancy	Early Preclinical	Magee-Women’s Research Inst. and Fndn., University of Pittsburgh (Pittsburgh, PA, USA)	Dapivirine Levonorgestrel	Hormonal	[69]
Tenofovir (TFV)/Efavirenz (EFV) Nanoparticles-in-film	HIV, HSV-1, HSV-2	Advanced Preclinical	Inst. For Research and Innovation in Health (i3S), University of Porto (Porto, Portugal)	Efavirenz (EFV) Tenofovir (TFV)	Non-Contraceptive	[53]
**Implants**						
Cabotegravir Pellet Implant with Levonorgestrel	HIV, Pregnancy	Early Preclinical	CONRAD (Norfolk, VA, USA)	Cabotegravir Levonorgestrel	Hormonal	[70]
Long-acting refillable nanofluidic implant (NanoMPI)- SC	HIV, Pregnancy	Early Preclinical	University of Washington (Seattle, WA, USA) Houston Methodist Hospital Research Institute (HMRI) (Houston, TX, USA)	Etonogestrel Islatravir (EFdA)	Hormonal	[71]
**Injectables (Subcutaneus)**						
Cabotegravir/Levonorgestrel Long-Acting MPT Injectable	HIV Pregnancy	Advanced Preclinical	CONRAD (Norfolk, VA, USA)	Cabotegravir Levonorgestrel	Hormonal	[70]
**Injectable In-situ Forming Implants**						
Ultra-Long-Acting MPT In situ Forming Implant (ISFI)	HIV Pregnancy	Early Preclinical	University of North Carolina (Chapel Hill, NC, USA)	Hormonal Contraceptive Antiretroviral (ARV)	Hormonal	[72]
**Gels (Vaginal/Rectal)**						
Yaso-GEL	HIV, Pregnancy, HSV-2, HPV, Gonorrhea	Advanced Preclinical	Yaso Therapeutics (Frisco, TX, USA)	Polyphenylene Carboxymethylene (PPCM)	Non-Hormonal	[73]
**Oral Tablet**						
Dual Prevention Pill (DPP) Regimen	HIV Pregnancy	Preclinical—Early (Pre1)	Population Council (New York City, NY, USA) Medicines360 (San Francisco, CA, USA)	Emtricitabine (FTC) Ethinyl Estradiol (EE) Levonorgestrel Tenofovir Alafenamide (TAF)	Hormonal	[74]
**Intravaginal Rings (Non-HIV)**						
Copper Intravaginal Ring (Cu-IVR)	Pregnancy, HSV-2, Zika virus	Early Preclinical	University of California (Davis, CA, USA)	Copper	Non-Hormonal	[75]

**Table 4:** The chemical structures of microbicides.

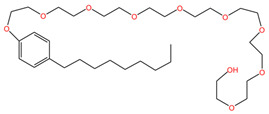	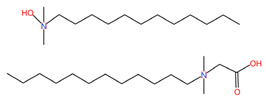
Nonoxynol-9 (C_33_H_60_O_10_)	C31G (C_30_H_64_N_2_O_3_)
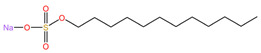	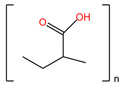
Sodium lauryl sulfate (C_12_H_25_SO_4_Na)	Carbopol 974P ([C_3_H_4_O_2_]_n_)
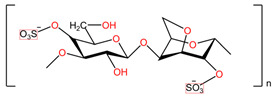	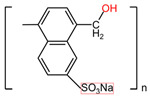
Ϳ-Carrageenans ([C_12_H_16_O_15_S_2_^2-^]_n_)	Naftalane sulfonate polymers ([C_11_H_9_NaSO_4_]_n_)
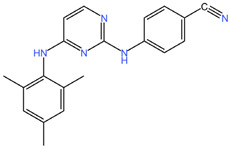	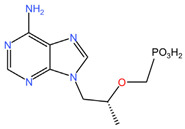
Dapivirine (C_20_H_19_N_5_)	Tenofovir (C_9_H_14_N_5_O_4_P)
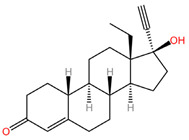	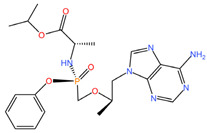
Levonorgestrel (C_21_H_28_O_2_)	Tenofovir alafenamide (C_21_H_29_N_6_O_5_P)
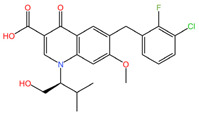	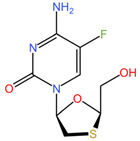
Elvitegravir (C_23_H_23_ClFNO_5_)	Emtricibatine (C_8_H_10_FN_3_O_3_S)
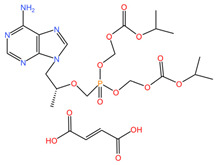	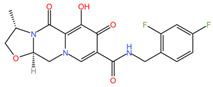
Tenofovir disoproxil fumarate (C_23_H_34_N_5_O_14_P)	Cabotegravir (C_19_H_17_F_2_N_3_O_5_)
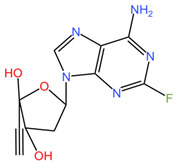	
Islatravir (C_12_H_12_FN_5_O_3_)	

## Data Availability

The data presented in this study are available upon request from the corresponding author.
